# Exon skipping for DMD

**DOI:** 10.1186/1750-1172-7-S2-A20

**Published:** 2012-11-22

**Authors:** Annemieke Aartsma-Rus, Jan JGM Verschuuren, Giles V Campion, Gert-jan B van Ommen, Judith CT van Deutekom

**Affiliations:** 1 Department of Human Genetics, Leiden University Medical Center, Leiden, the Netherlands; 2 Department of Neurology, Leiden University Medical Center, Leiden, the Netherlands; 3 Prosensa Therapeutics B.V., Leiden, the Netherlands

## 

Duchenne muscular dystrophy (DMD) is a severe, progressive muscle-wasting disorder, while Becker muscular dystrophy (BMD) is milder muscle disease [1]. Both are caused by mutations in dystrophin, a protein, which stabilizes muscle fibers during contraction by linking muscle actin to the extracellular matrix. In DMD patients mutations disrupt the open reading frame, generating prematurely truncated, nonfunctional dystrophins [2]. In BMD patients, mutations maintain the reading frame allowing production of internally deleted, partly functional dystrophins.

The exon skipping approach uses antisense oligonucleotides (AONs) to induce skipping of targeted exons during pre-mRNA splicing, with the aim of reading frame restoration, converting of the severe DMD into the milder BMD phenotype [3]. This approach is mutation specific. However, as mutations cluster in a few hotspots, skipping of some exons applies to larger groups of patients (e.g. exon 51 skipping applies to 13%) [4].

After promising results in cultured cells and animal models where AON treatment allowed in dystrophin restoration (reviewed in [3]), a first clinical trial was performed by LUMC and Prosensa Therapeutics, where four DMD patients where intramuscularly injected with an exon 51 (GSK2402968/PRO051, a 2’-O-methyl phosphorothioate (2OMePS) AON) [5]. Exon skipping and dystrophin restoration was observed for each patient in muscle biopsies taken 4 weeks after the injection.

Towards systemic application, studies in animal models revealed that dystrophic muscles facilitated uptake of 2OMePS AONs and that subcutaneous delivery was feasible [6]. In a subsequent clinical trial, patients were subcutaneously injected with 2OMePS AONS targeting exon 51 [7]. Dystrophin was restored in a dose-dependent manner at levels up to 15%. All patients were enrolled in an open label extension study and have received subcutaneous AON injections at 6 mg/kg for over 2.5 years. A pivotal, double-blind, placebo-controlled multicenter trial for exon 51 skipping is currently ongoing (coordinated by GlaxoSmithKline).

In parallel, preclinical studies to further optimise treatment regimens are in progress as well as clinical trials for additional exons for exon 44 skipping (PRO044, applicable to 6% of patients). Trials are planned for exon 45 and 53 skipping (PRO045 and PRO053, both applicable to 8% of patients).

The mutation specificity of the approach poses challenges to drug development regulations. A concerted effort of academic researchers, industry, regulators and patients is needed to adapt regulations to enable application of these personalised medicine approaches to rare diseases.
